# Isolation and characterization of *GmMYBJ3*, an R2R3-MYB transcription factor that affects isoflavonoids biosynthesis in soybean

**DOI:** 10.1371/journal.pone.0179990

**Published:** 2017-06-27

**Authors:** Mingzhu Zhao, Tianliang Wang, Ping Wu, Wenyun Guo, Liantai Su, Ying Wang, Yajing Liu, Fan Yan, Qingyu Wang

**Affiliations:** Jilin Key Laboratory for Crop Genetic Engineering, College of Plant Science, Jilin University, Changchun, China; Institute of Genetics and Developmental Biology Chinese Academy of Sciences, CHINA

## Abstract

Isoflavonoids are secondary metabolites that play a variety of roles in plant-microbe interactions and plant defenses against abiotic stresses. Here we report a new MYB transcription factor (TF) gene, *GmMYBJ3*, that is involved in the isoflavonoids biosynthesis. The *GmMYBJ3* gene is 1,002 bp long and encodes a protein of 333 amino acids. Amino acid sequence analysis showed that *GmMYBJ3* is a typical R2R3 MYB TF. Yeast expression experiment demonstrated that *GmMYBJ3* has its transcription activity in the nucleus and is transiently expressed in onion epidermal cells. The *GmMYBJ3* gene was transformed into soybean and the expression activity of the *GmMYBJ3* gene was significantly positively correlated with total isoflavonoid accumulation in soybean. Transient expression assays indicated that *GmMYBJ3* can activate *CHS8* expression. Furthermore, we analyzed the expressions of several genes known involved in the isoflavonoid biosynthesis, including *CHS8*, *CHI1A*, *PAL1*, *IFS2* and *F3H*, in the *GmMYBJ3* transgenic plants. The results showed that the expression levels of *CHS8* and *CHI1A* were significantly increased in the transgenic plants compared to wild-type plants, but those of *PAL1*, *IFS2* and *F3H* remained similar between the transgenic and wild-type plants. These results suggest that *GmMYBJ3* participates in the isoflavonoid biosynthesis through regulation of *CHS8* and *CHI1A* in soybean.

## 1. Introduction

Isoflavonoids are a group of plant natural secondary metabolites. Isoflavonoids are mainly found in the Papilionideae family, and are abundant in soybean, *Glycine max* (L.) Merr., and other legume species. They play a variety of roles in plant-microbe interactions, such as functioning as signal molecules for symbiosis between soybean and *Bradyrhizobium japonica* [1,2]. They also serve as phytoalexins in response to pathogen attacks [3,4] and abiotic stresses such as UV irradiation and drought [5–7]. Interest in these compounds has recently increased because they are associated with important preventive and therapeutic medicinal properties [8,9].

Isoflavonoids are synthesized through a branch of the phenylpropanoid pathway present in legumes. Chalcone establishes the first step in the branched pathway for the synthesis of flavonoids and isoflavonoids [10] and is generated in a reaction catalyzed by chalcone synthase (CHS). CHS is encoded by a single gene in *Arabidopsis thaliana*, but there are multiple copies in other plants, including petunia and soybean [11,12]. The soybean genome contains a gene family consisting of nine *CHS* gene members, designated from *CHS1* to *CHS9*, with *CHS1* having a duplicate [13]. Comparative gene expression analysis between soybean cultivars with contrasting seed isoflavonoid contents revealed a critical role of the *CHS7* and *CHS8* genes in isoflavonoid biosynthesis [14]. RNAi silencing of the *CHS8* gene reduced the level of isoflavonoids in soybean hairy roots, providing a line of evidence that *CHS8* is involved in regulation of isoflavonoid biosynthesis [15]. In the isoflavonoid biosynthesis, isoflavonoid synthase (IFS) is also necessary. The first key step of isoflavonoid biosynthesis is liquiritigenin and naringenin conversion to daidzein or genistein, which is catalyzed by *IFS*. Two *IFS* genes were identified in soybean, *IFS1* and *IFS2*, and there are 14 amino acids that differ between their protein products. The expression of *IFS1* in *A*. *thaliana* can induce the production of the isoflavone genistein in this non-legume plant [16].

The phenylpropanoid pathways are predominantly regulated at the transcriptional level by members of transcription factors, especially the MYB TFs [17–19]. Genetic markers linked to controlling corresponding quantitative traits may be used to select for favorable alleles effectively, and was verified in some agronomic traits [20,21]. Therefore, we focus in this study on the MYB TFs that are mapped in the soybean isoflavonoid QTL. Even though interaction exists between MYB TFs and other factors to regulate several branches of the phenylpropanoid pathways, MYB proteins are believed to be the key components [18]. For example, *GmMYB176* affects isoflavonoid biosynthesis in soybean hairy roots through interaction with 14-3-3 protein in the nucleus [22]. The genes required for the synthesis of flavonols and anthocyanins are induced by maize *MYB C1* in tomato, except for *F3′H*, *F3′5′H* and *CHI* [23]. Introduction of CRC (a chimeric transcription factor of maize C1 and R) into soybean resulted in a significant increase in seed isoflavonoid levels [24]. *GmMYB39* down regulates the isoflavonoid biosynthesis in soybean [25]. MYB134 regulates proanthocyanidin synthesis in poplar [26]. Overexpression of *AtMYB12* in *Arabidopsis* displays a flavonol accumulation in transgenic lines [27].

In this study, we compared the isoflavonoid QTLs previously mapped [28–30] and found a common isoflavonoid QTL mapped with different populations in different environments. Only one R2R3 MYB TF, *GmMYBJ3*, was found in this common QTL, suggesting that it is likely a candidate gene for isoflavonoid biosynthesis. We associated the expression activity of *GmMYBJ3* with the isoflavonoid content in soybean. Subcellular localization assays in onion epidermal cells indicated that *GmMYBJ3* encodes a nucleus-localized protein and yeast hybrid analysis showed that it has transcriptional activity. Transient expression assays showed that *GmMYBJ3* could activate *CHS8* expression. Overexpression of *GmMYBJ3* in the soybean transgenic plants up-regulates the expression level of *CHS8* and *CHI1A* and increases the total isoflavonoid content.

## 2. Results

### 2.1. Isolation and sequence analysis of *GmMYBJ3*

**Fig 1:**
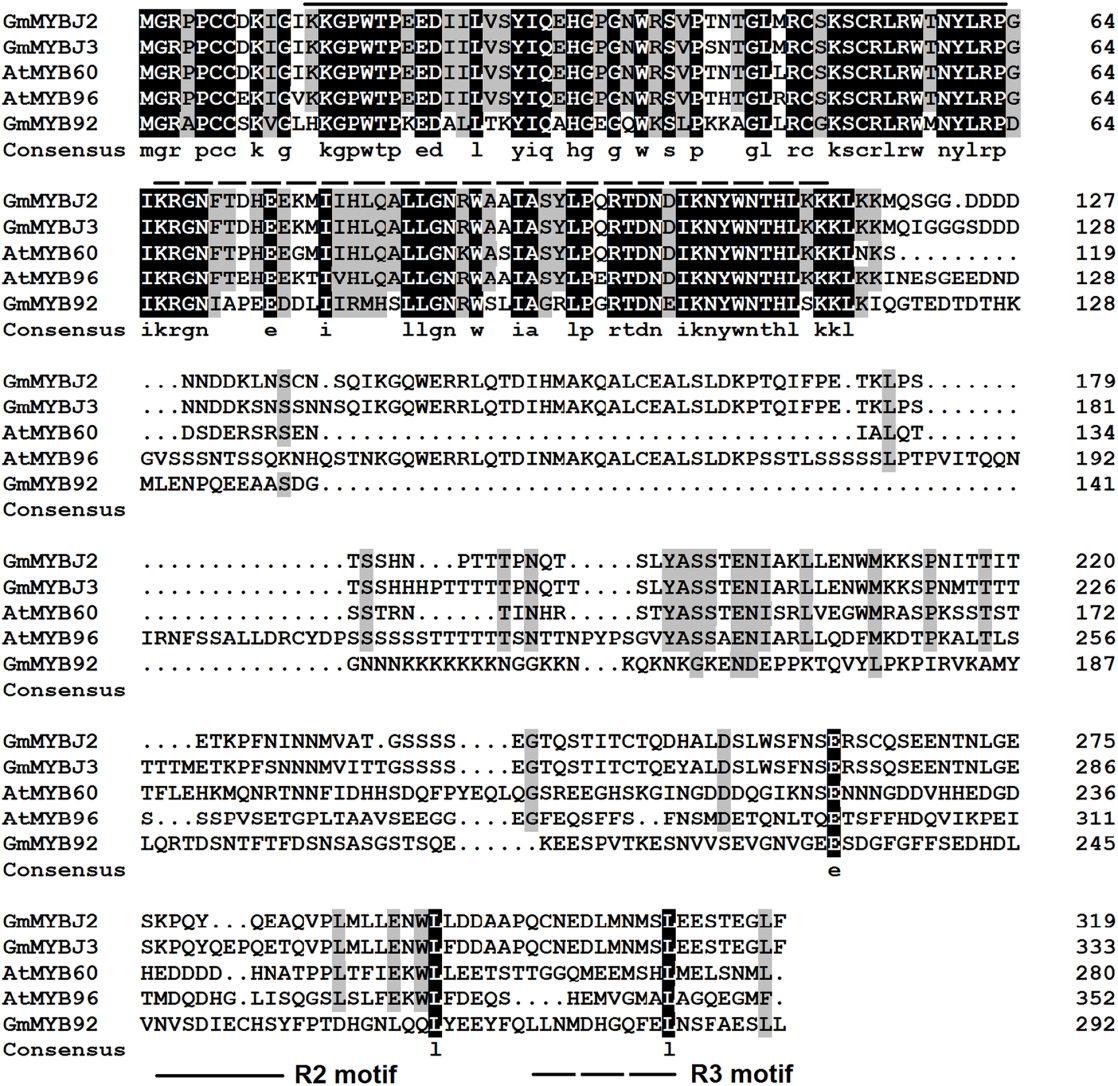
Alignments of the deduced amino acid sequences of *GmMYBJ3* with those of MYB TFs identified from other plant species. Identical and similar amino acid residues are shown in black and gray, respectively. *Gm*, *Glycine max; At*, *Arabidopsis thaliana*. The GenBank accession numbers of the genes are: *GmMYBJ2*, KC759158.1; *GmMYBJ3*, KU664645; *AtMYB60*, AF062895.1; *AtMYB96*, NP_201053.2; *GmMYB92*, DQ822903.1.

**Fig 2:**
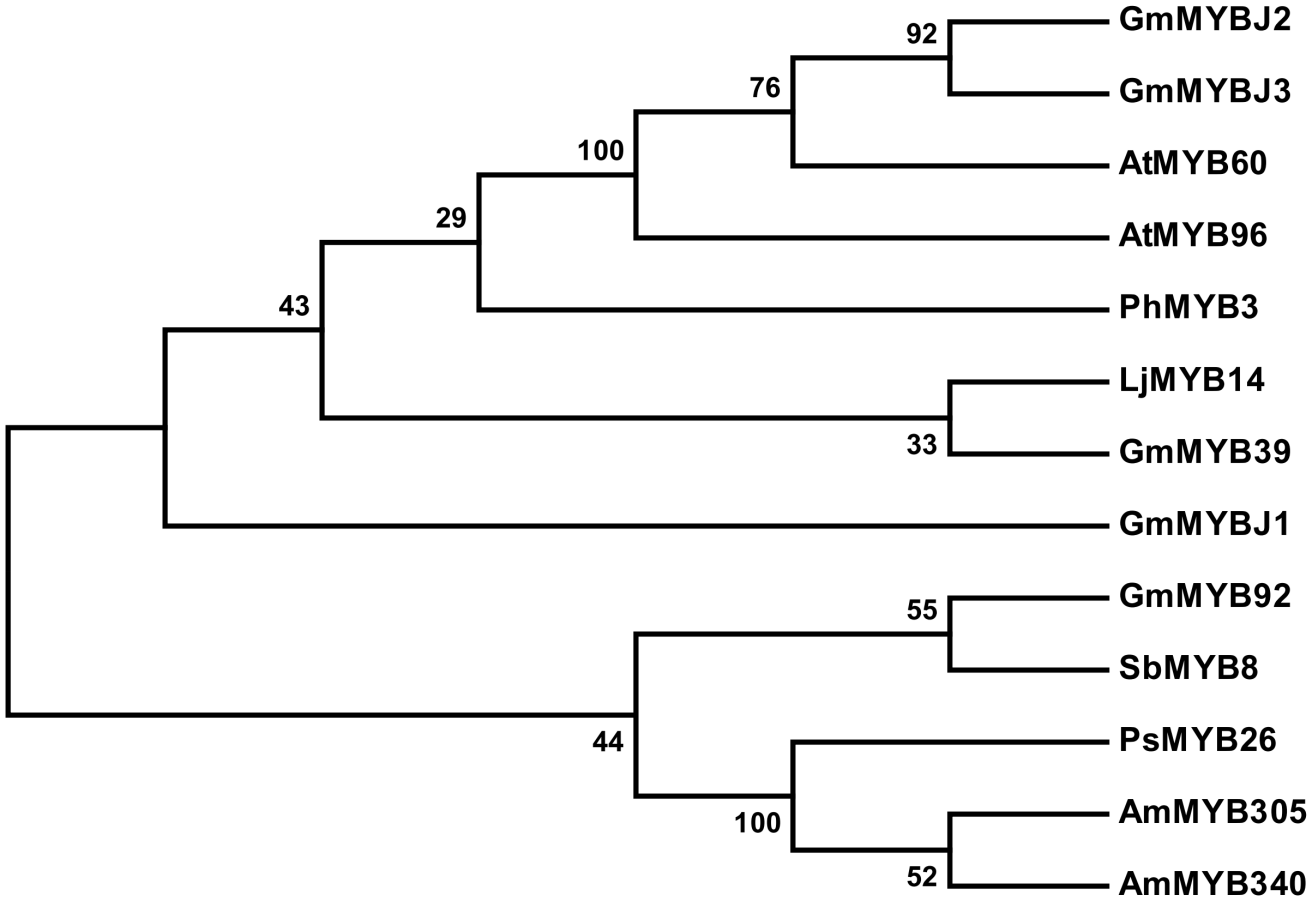
Phylogenetic analysis of *GmMYBJ3* with other R2R3-MYB TFs. The tree was constructed using MEGA 7.0 based on the R2 and R3 motifs of the R2R3-MYB TFs. The numbers at the nodes indicate the percentages of bootstrap values from 1000 replications. *Gm*, *Glycine max; Ps*, *Pisum sativum; Am*, *Antirrhinum majus; At*, *Arabidopsis thaliana; Ph*, *Petunia hybrid; Lj*, *Lotus japonicas*. The GenBank accession numbers of the genes are: *GmMYBJ3*, KU664645; *GmMYBJ2*, KC759158.1; *GmMYBJ1*, KC751453.1; *GmMYB92*, DQ822903.1; *AtMYB60*, AF062895.1; *SbMYB8*, KF008657.1; *PsMYB26*, Y11105.1; *AtMYB96*, NP_201053.2; *PhMYB3*, Z13998.1; *AmMYB305*, P81391.1; *AmMYB340*, P81396.1; *LjMYB14* [34]; *GmMYB39*, XP_003538653.3.

Two adjacent QTLs, *GLY1* and *qGm06*, were previously mapped to Chromosome 6 that control soybean isoflavonoid content [31,32]. *GLY1* were mapped to a region closely linked with SNP marker BARC-031337-07051 using a population consisting of 274 F5:8 RILs (recombining inbred lines) derived from Essex × Williams 82 and grown at three locations (Knoxville, TN; Harrisburg, IL; and Stuttgart, AR). The SNP of BARC-031337-07051 was physically mapped to the 16679945 position of the chromosome 6 (http://soybase.org/). The QTL *qGm06* was mapped to an interval between BARC-063661-18416 and BARC-066175-19800 using a population consisting of 188 F7 RILs derived from Magellan × PI 437654 and grown at two locations (the University of Missouri Bradford Research and Extension Center, and the University of Missouri Delta Research Center). The SNP of BARC-063661-18416 was physically mapped to the 17230167 position and that of BARC-066175-19800 to the 18736894 position of Chromosome 6 (http://soybase.org/). Therefore, the region between BARC-031337-07051 and BARC-066175-19800, physically spanning from the 16679945 position to the 18736894 position, contains at least one of the two QTLs controlling soybean isoflavonoid content. Therefore, we searched this region carefully and found only one MYB transcription factor (TF) and the gene ID of this MYB TF is Glyma.06g193600 (Version: Glyma2.0). We designated this gene as *GmMYBJ3*. *GmMYBJ3* has a full-length ORF of 1002 bp and was predicted to encode a protein of 333 amino acid residues with a calculated mass of 37.7 kDa and a pI of 5.81. The CDD v3.15 (https://www.ncbi.nlm.nih.gov/Structure/cdd/wrpsb.cgi) was used to search for the conserved domains located at amino termini and responsible for binding to DNA sequence. As shown in Fig 1, there are two MYB imperfect repeats, R2 and R3, which consist of 50 (13–63) and 48 (66–114) amino acids, respectively. Phylogenetic analysis (Fig 2) showed that *GmMYBJ3* clustered with *Arobidopsis AtMYB60* that regulates anthocyanin biosynthesis in lettuce [33]. Both anthocyanin and isoflavonoid are products of the phenylpropanoid metabolic pathway. Because *GmMYBJ3* was found from the soybean isoflavonoid QTL region, it is homologous to *AtMYB60*, and its R2R3 participates in regulating the phenylpropanoid pathway, we hypothesized that *GmMYBJ3* is involved in the transcriptional regulation of isoflavonoid biosynthesis.

### 2.2 Subcellular localization of the *GmMYBJ3* protein

**Fig 3:**
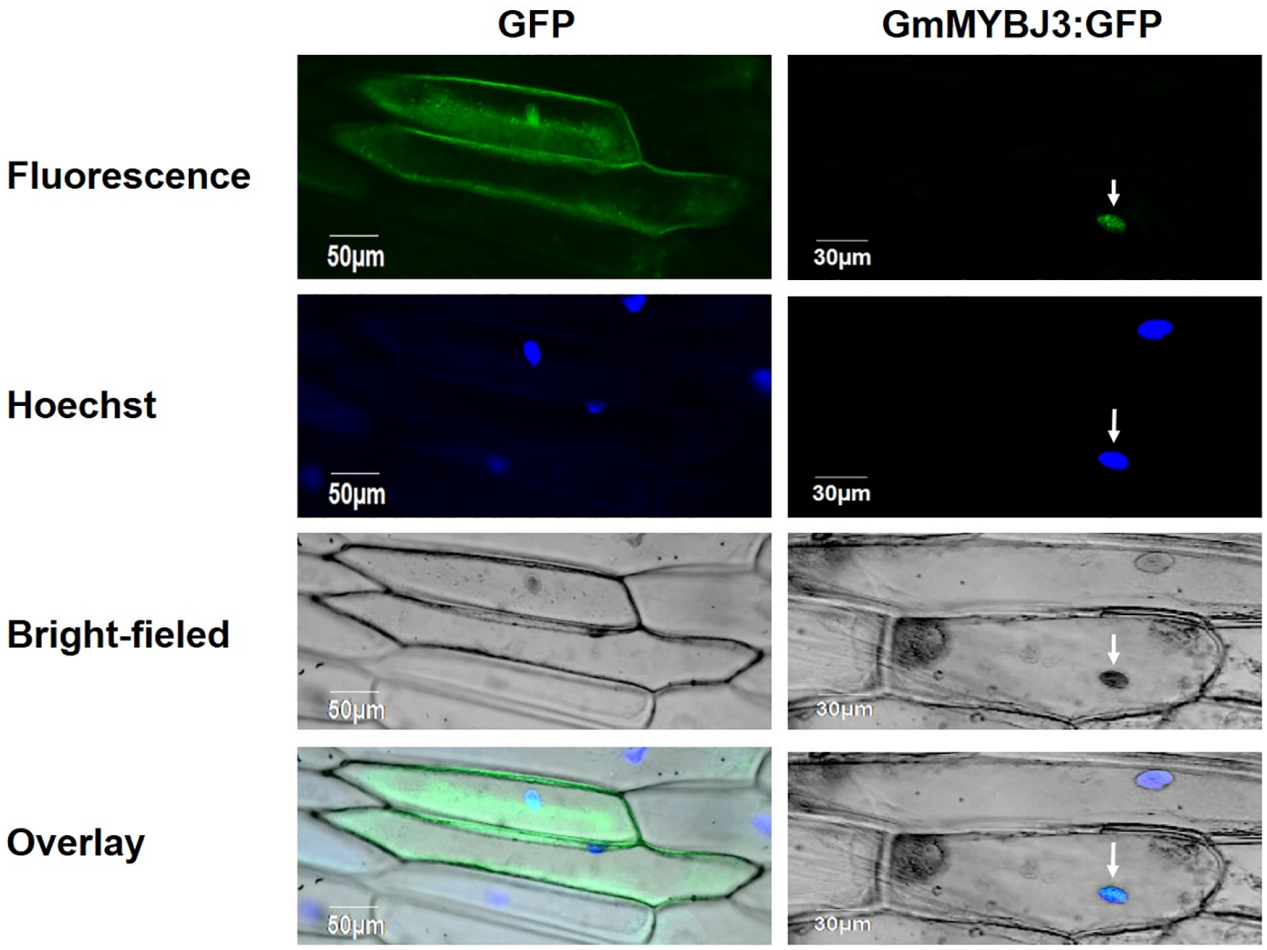
Subcellular localization of the *GmMYBJ3*:GFP fusion protein in onion epidermal cells. Onion epidermal cells transformed with GFP were used as control and the results were visualized by confocal microscopy. The nucleus of cell was indicated by arrow.

The *GmMYBJ3* protein was localized in the nucleus by online prediction (http://www.csbio.sjtu.edu.cn/bioinf/plant/). To further confirm this localization result of the *GmMYBJ3* protein, we deleted its stop codon and fused its ORF to the N terminus of GFP that is under control of the CaMV 35S promoter. The pBI121 constructs containing *GmMYBJ3*:GFP and containing GFP alone were transformed into onion epidermal cells, respectively, through *Agrobacterium* EHA105-mediated transient expression. The results showed that the *GmMYBJ3*:GFP fusion protein resided in the nucleus of the cells, which was consistent with the online predicted result, but the control construct containing GFP alone was visualized throughout the cells (Fig 3).

### 2.3 Transcriptional activation of *GmMYBJ3 in vivo*

**Fig 4:**
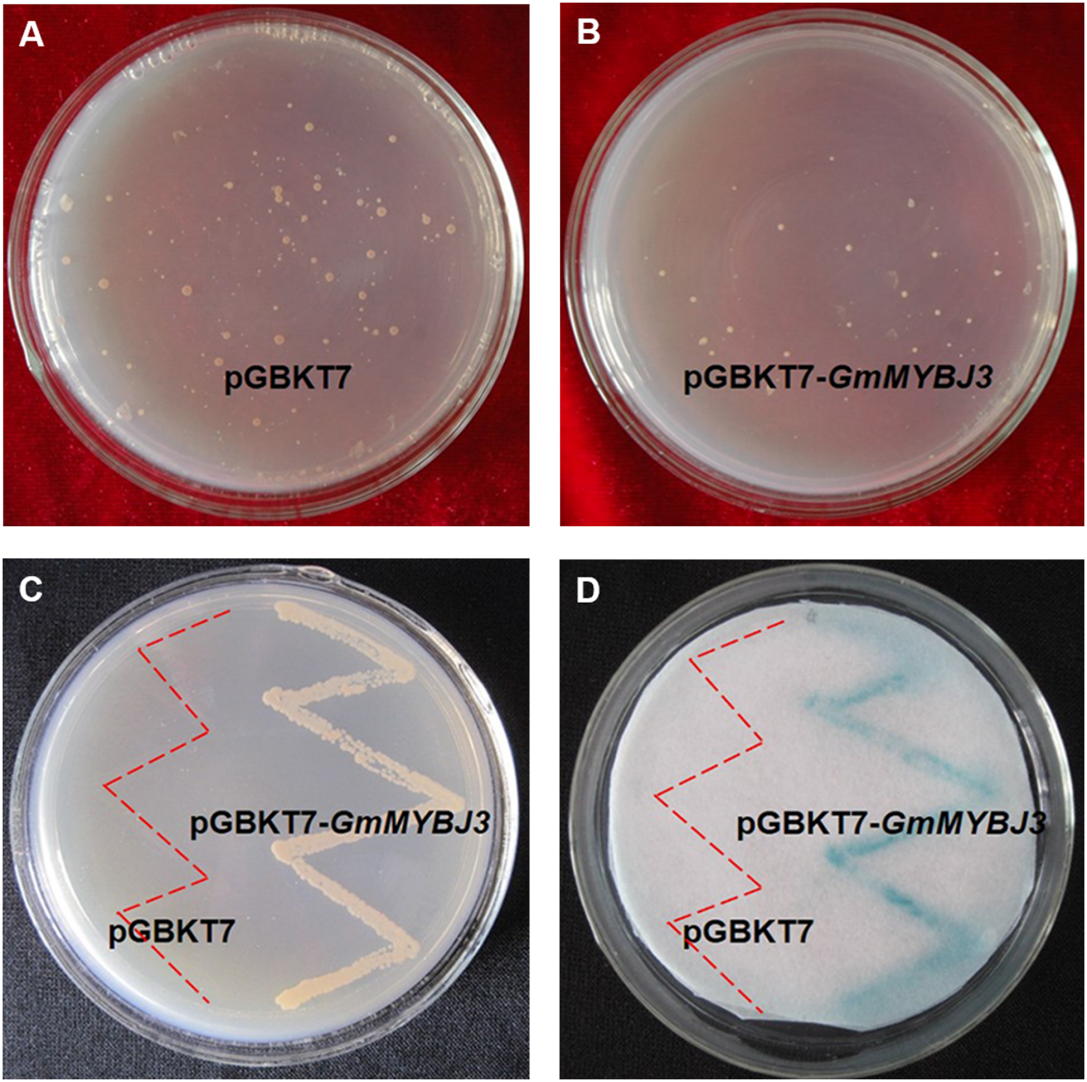
Transcription activation analysis of the *GmMYBJ3* protein. (A and B) Yeasts containing the pGBKT7 vector only and the pGBKT7-*GmMYBJ3* construct screened on the solid medium SD/-Trp. (C) Yeasts containing the pGBKT7 vector only and the pGBKT7-*GmMYBJ3* construct grown on the solid medium SD/-Trp/-His/-Ade with 10 mM 3-AT. (D) 5-bromo-4-chloro-3-indolyl β-D-galactopyranoside activation detection of transformed yeast thalli. The red dashed lines in C and D represent the regions where the yeasts containing the pGBKT7 vector only were streaked.

The transcriptional activity of *GmMYBJ3* was tested using the pGBKT7 vector that expresses proteins fused to the GAL4 DNA binding domain from the constitutive *ADH1* promoter in the yeast GAL4 system (PT3248-5, Clontech, USA). The transcriptional activator protein would activate the *HIS* and *lacZ* reporter genes, thus allowing yeast to survive on the histidine-deficient medium and showing color in the β-galactosidase assay. The fusion plasmid pGBKT7-*GmMYBJ3* and the empty vector pGBKT7 were transformed into yeast AH109 cells, respectively, and screened on the solid medium SD/-Trp so that the positive transformants will survive (Fig 4A and 4B). To further characterize the positive transformants, we streaked the transformants on the solid medium SD/-Trp/-His/-Ade plus 3-AT. The fusion plasmid cells survived on the SD/-Trp/-His/-Ade plus 3-AT plate, but the pGBKT7 control plasmid cells did not. This result indicated that the fusion effectors were expressed and able to activate the expression of the *HIS* reporter gene (Fig 4C). For the 5-bromo-4-chloro-3-indolyl β-D-galactopyranoside activation assay, strong blue signals were observed for the pGBKT7-*GmMYBJ3* cells, reflecting that the *lacZ* reporter gene was activated, whereas no colorful signal was detected for the pGBKT7 cells, indicating that no transcriptional activation occurred (Fig 4D).

### 2.4 *GmMYBJ3* activates the transcription of *CHS8 in vivo*

**Fig 5:**
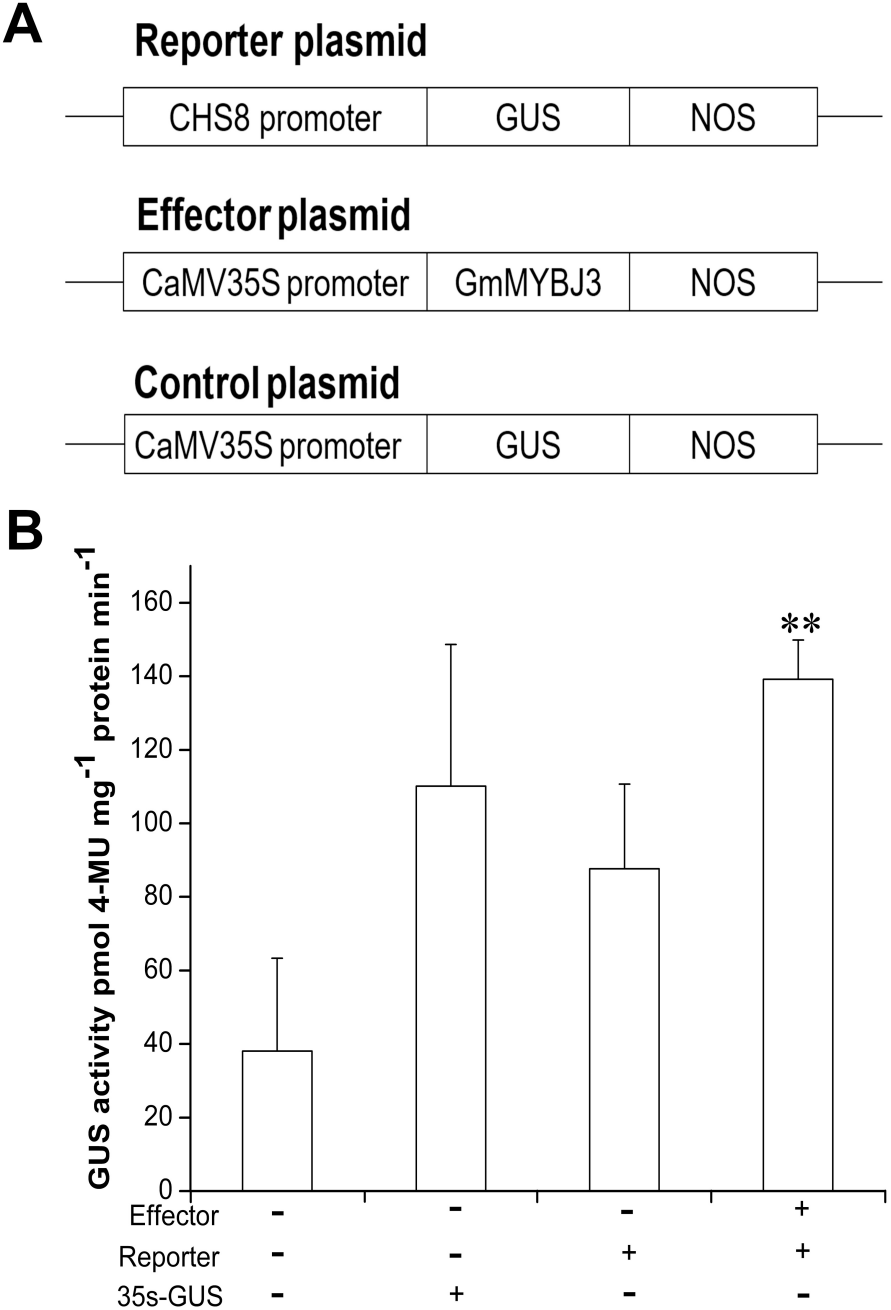
Transcriptional activation analysis of *GmMYBJ3* in tobacco leaves. (A) Schematic diagrams of the reporter, effector and control plasmid constructs used for the transient expression assays. (B) Transcriptional activation analyzed by GUS activity assay. The data are presented as the averages of three independent assays ± SD. ** represents significant difference at *P* ≤ 0.01 between the CHS8pro:GUS construct and the CHS8pro:GUS construct co-transfected with the effector plasmid. +,–represent whether the reporter and/or effecter plasmids were co-transfected or not.

To find whether *GmMYBJ3* activates *CHS8* expression in soybean, we performed a transient expression experiment using tobacco leaves. A CHS8pro:GUS fusion reporter construct, an effector plasmid and a control plasmid were constructed (Fig 5A). The GUS activity of the 35Spro:GUS construct without the effector was higher than the GUS activity of the transformed CHS8pro:GUS construct in tobacco leaves. However, the co-transfection experiment with the effector and CHS8pro:GUS reporter constructs showed that the GUS activity was 1.26-fold higher than that of the 35Spro:GUS control plasmid and was 1.59-fold higher than that of the CHS8pro:GUS reporter plasmid. The GUS activity was significantly different between the CHS8pro:GUS construct and the CHS8pro:GUS construct co-transfected with the effector plasmid. From the results of the transient expression assay (Fig 5B), we noted that *GmMYBJ3* highly likely trans-activated CHS8pro:GUS expression.

### 2.5 *GmMYBJ3* is expressed in the tissues where isoflavonoids are synthesized in soybean

**Fig 6:**
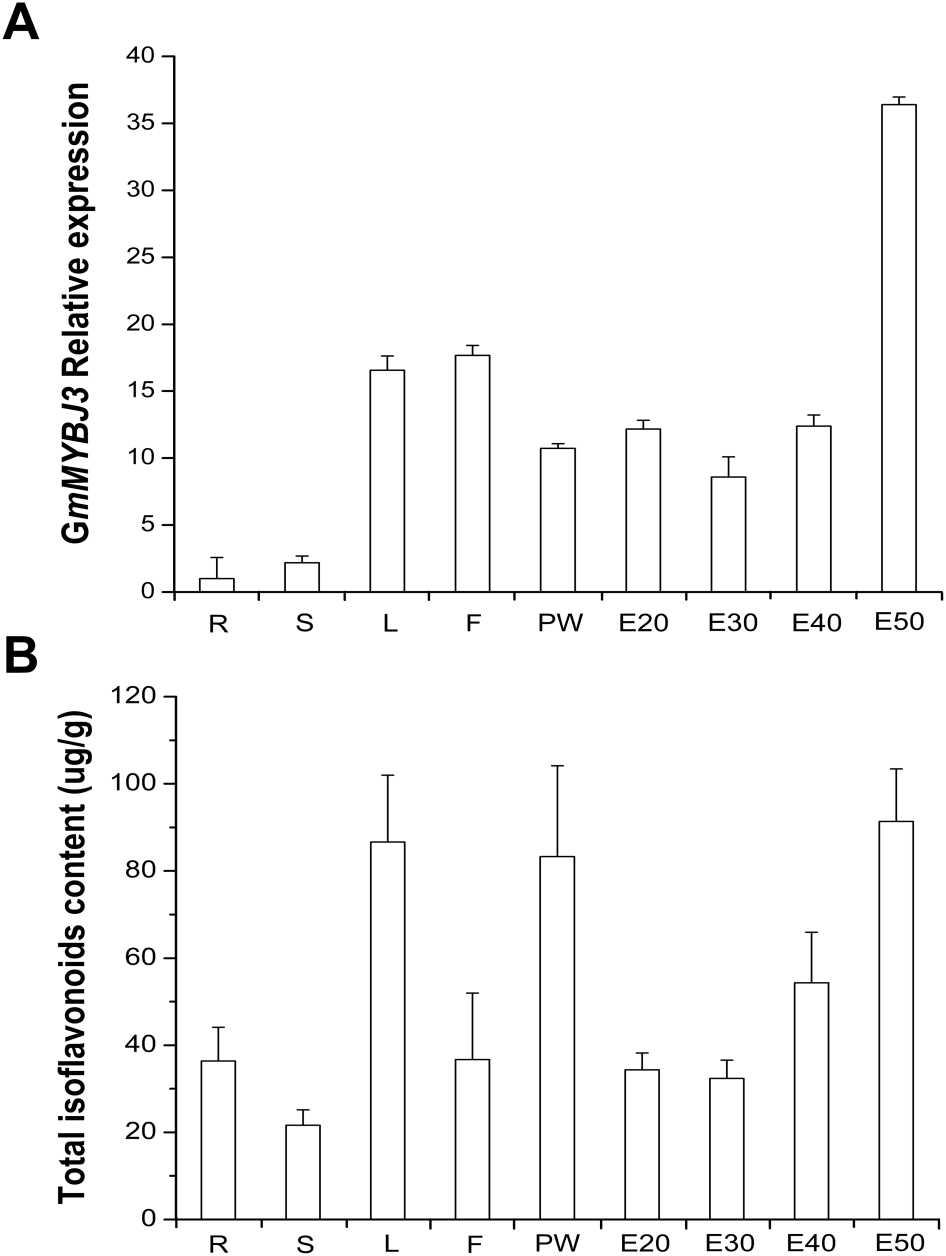
*GmMYBJ3* expression and isoflavonoids accumulation. (A) Expressions of *GmMYBJ3* in different tissues of soybean cv. Williams 82 determined by quantitative RT-PCR. The tissues are roots (R), stem (S), leaf (L), flower (F), pod wall (PW), and embryos collected at 20 days after pollination (E20), 30 days after pollination (E30), 40 days after pollination (E40) and 50 days after pollination (E50). (B) Total isoflavonoid content analysis in these tissues by HPLC. In both (A) and (B), the data are the mean ± SD of samples in three independent plants, with three technical replicates for each plant.

To confirm that *GmMYBJ3* expresses in tissues where isoflavonoids accumulate, we investigated the expression of *GmMYBJ3* in various tissues of soybean (Fig 6A) by quantitative RT-PCR and analyzed the correlation between its expression level and isoflavonoids content. The results showed that *GmMYBJ3* expressed in all the tissues studied, but its expression level increased as embryos developed. Fig 6B shows the contents of isoflavonoids in tissues. Correlatioin analysis showed that the *GmMYBJ3* expression level positively correlated with the total isoflavonoids accumulation in soybean (Pearson’s *r* = 0.674, *P* ≤ 0.05) (S1 Table). These results indicated that the expression activity of *GmMYBJ3* was associated with the synthesis of isoflavonoids.

### 2.6 Overexpression of *GmMYBJ3* enhances isoflavonoid content in transgenic soybean

**Fig 7:**
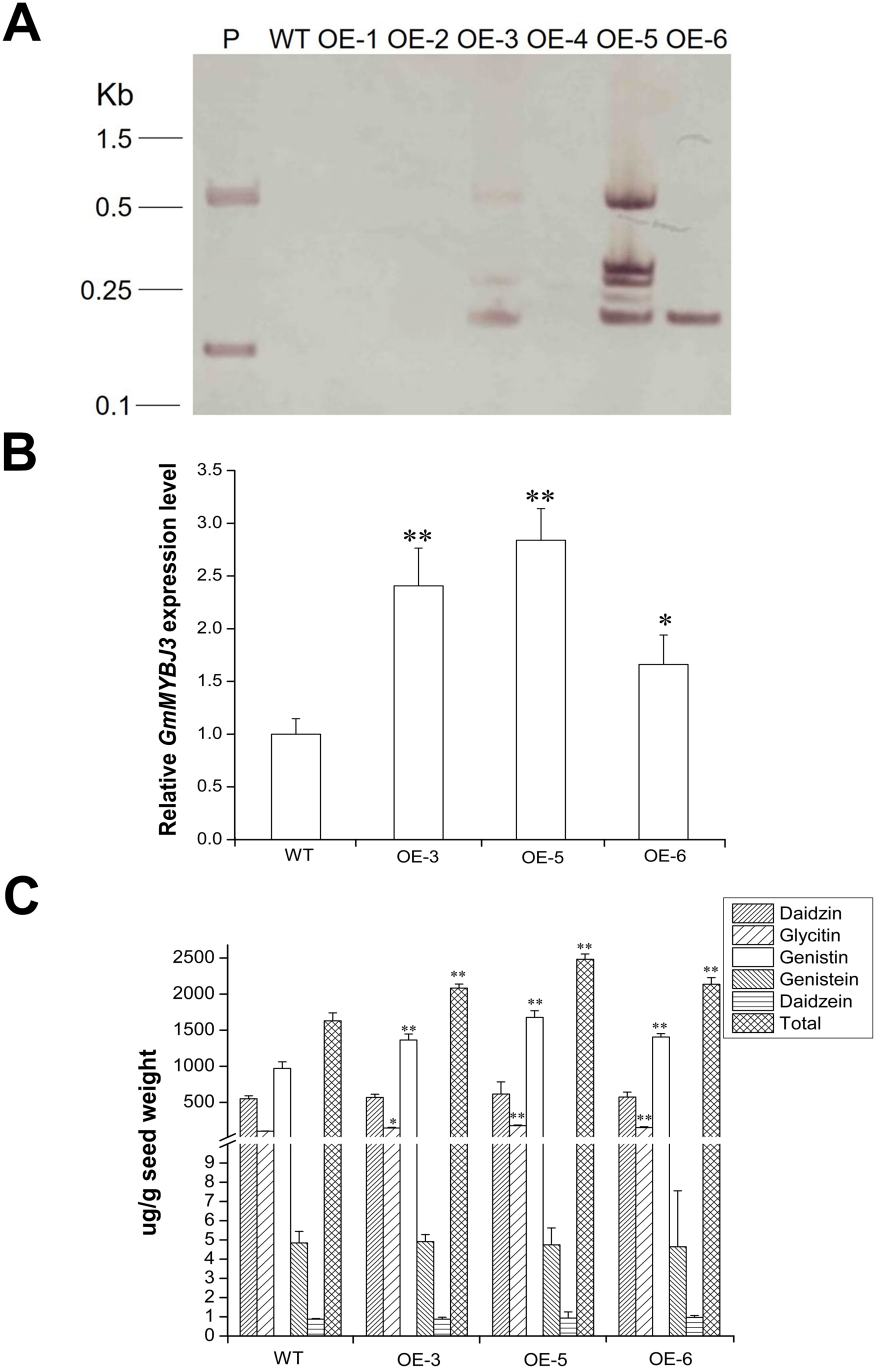
Genetic transformation and analysis of *GmMYBJ3* in soybean. (A) Southern blot analysis of T_2_ transgenic plants using the bar gene fragment as a probe. The DNAs of transgenic plants were digested with *Hind* III. The bands present in the transgenic lines, but undetected in the wild type were used to define the positive transgenic plants. P, pCB35SR1R2-GFP-*GmMYBJ3* vector DNA used as a positive control; WT, wild type; OE, *GmMYBJ3* overexpression lines, with 1–6 representing different transgenic lines. (B) Quantitative RT-PCR analysis showing the expression level of *GmMYBJ3* in the seeds of the positive T_2_ transgenic plants compared to the wild type, Jilin 35 (control). Data represent the mean ± SD of three independent seeds, with each plant having three technical replicates. * and ** represent significant differences at *P* ≤ 0.05 and 0.01 between the transgenic lines and the WT. (C) Isoflavonoid content in the seeds of the positive T_2_ transgenic compared to the WT control. The data of wild type (control) represent the mean ± SD of three independent plants, with each plant having three technical replicates. The data for each of the independent positive transgenic lines are the means of three technical replicates, and the error bars show SEM. * and ** represent significant difference at *P* ≤ 0.05 and 0.01 between the transgenic lines and the WT.

To demonstrate the function of *GmMYBJ3* in isoflavonoid biosynthesis, the overexpression of *GmMYBJ3* was performed using cv. Jilin 35 as the host plant through the *Agrobacterium*-mediated transformation of soybean embryonic tips (unpublished). The T_0_ transgenic plants were selected on the medium containing Basta. Southern blots were prepared from the leaves of independent T_2_ plants and hybridized to confirm the transgenic plants. The results showed that *GmMYBJ3* was transformed into Jilin 35 cultivar (Fig 7A). Quantitative RT-PCR with the positive T_2_ transgenic plants showed that the expression level of *GmMYBJ3* was significantly increased in the transgenic plants compared to the WT control (Fig 7B) and the isoflavonoids content was measured by HPLC (Fig 7C). HPLC analysis showed that the glycitin, genistin and total isoflavonoid contents were significantly increased in the positive transgenic plants compared to the WT control, but the contents of daidzin, genistein and daidzein largely remained unchanged.

### 2.7 Expression of the genes known to be involved in the isoflavonoid biosynthesis in the seeds of transgenic plants

**Fig 8:**
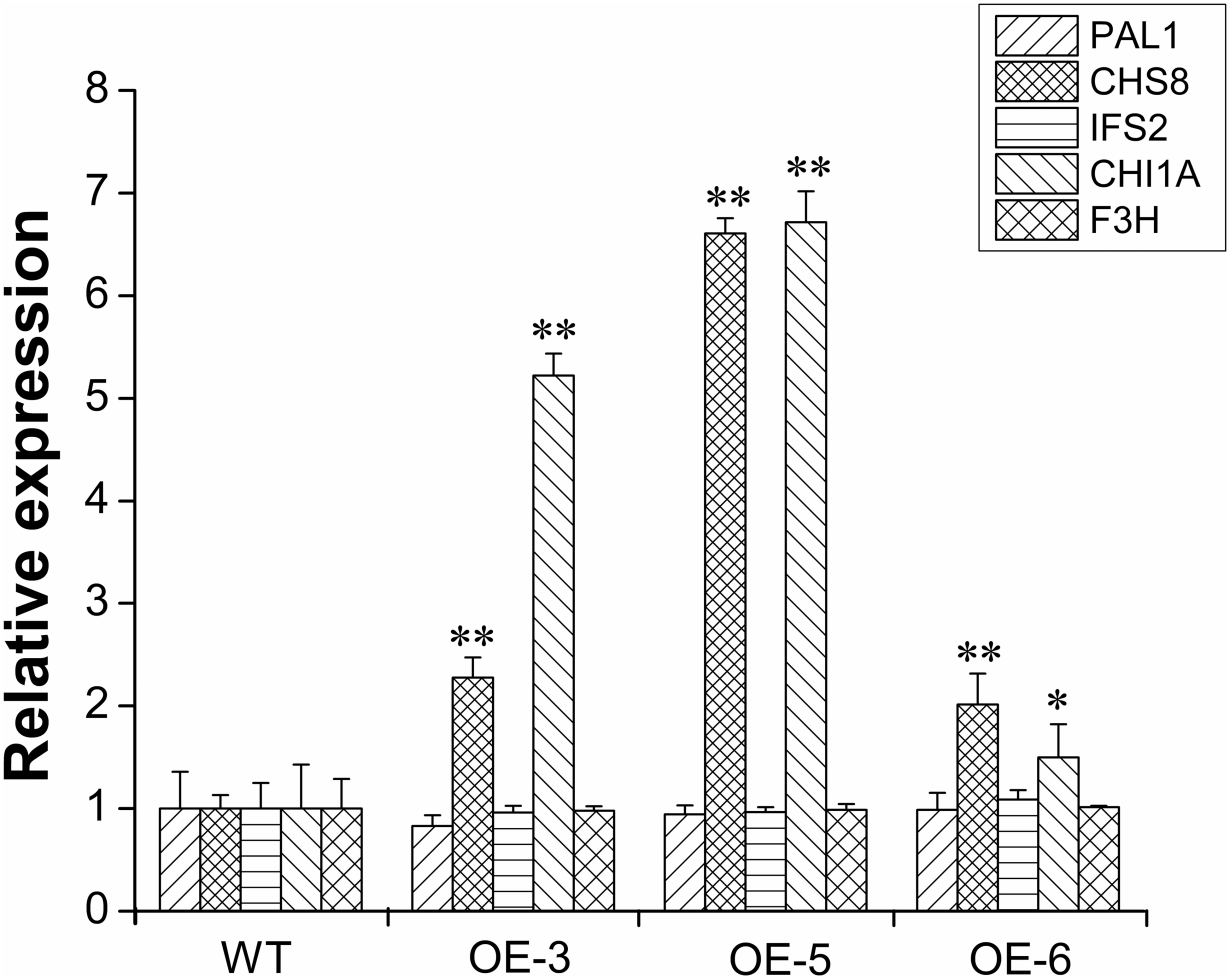
Quantitative RT-PCR analysis of the key genes, *PAL1*, *CHS8*, *IFS2*, *CHI1A* and *F3H*, involved in the isoflavonoids synthesis in the seeds of positive T_2_ transgenic lines. WT, wild type; OE, independent *GmMYBJ3* overexpression lines. The expression data of transgenic and wild-type lines represent the mean ± SD of three independent plants, with each plant having three technical replicates. * and ** represent significant differences at *P* ≤ 0.05 and 0.01, respectively, between the transgenic lines and WT.

The enzymes, *PAL1* (phenylalanine ammonia-lyase), *CHS8* (chalcone synthase), *IFS2* (isoflavone synthase) and *CHI1A* (chalcone isomerase), are known to catalyze the biosynthesis of isoflavonoids in soybean seeds [14,15,35], and *F3H* (flavanone-3-hydroxylase) is a competitive enzyme for anthocyanin synthesis in a branched fashion. Therefore, we further analyzed the expressions of the genes encoding these enzymes in the T_2_ transgenic plants to infer the role of *GmMYBJ3* in the isoflavonoids biosynthesis in soybean. The results showed that the *CHS8* and *CHI1A* genes were significantly up-regulated in the seeds of the T_2_ transgenic plants. However, the expression levels of *PAL1*, *IFS2* and *F3H* underwent little changes compared to the WT plants (Fig 8). These changes suggested that *GmMYBJ3* may be a regulator of the isoflavonoid biosynthesis pathway in soybean seeds.

## 3. Discussion

The MYB TFs, especially the group of R2R3-MYB, are known to be involved in the regulation of the phenylpropanoid metabolic pathway [36]. In this study, we have identified a MYB transcription factor from soybean, designated as *GmMYBJ3*, and characterized it in its functions in the phenylpropanoid metabolic pathway. Analysis of the *GmMYBJ3* gene indicates that it is a typical R2R3-MYB with conserved R2 and R3 motif, and gene phylogenetic analysis shows that it clusters with *GmMYBJ2*, and is also homologous to *AtMYB60*. Overexpression of *GmMYBJ2* enhances stress tolerance in *Arabidopsis thaliana* [37]. *AtMYB60* regulates stomatal movements, plant drought tolerance [38] and anthocyanin biosynthesis in lettuce [33]. Pleiotropy is very common in MYB TF [39], and *GmMYBJ3* may play different roles in different physiological and biochemical processes. Because *GmMYBJ3* was identified from a genomic region containing a soybean isoflavonoid QTL, we speculate that *GmMYBJ3* may be involved in regulating the isoflavonoid biosynthesis. The sub-localization of *GmMYBJ3* reveals that it is a nuclear localization protein, which is in agreement with its role as a transcription factor. Most R2R3 MYB TFs are presumed to be transcriptional activators with activation domains (ADs) in the C-terminal region [40], but it is known that the N-termini of MYB TFs are conserved. One yeast hybrid analysis has confirmed that the *GmMYBJ3* gene has a transcription activation domain, even though further tests in the plant system remain.

Analysis of gene expressions during soybean embryo development revealed the crucial roles of the *CHS7* and *CHS8* genes in isoflavonoid biosynthesis [14]. Further studies of the *CHS7* and *CHS8* promoters have indicated that *CHS8* is involved in isoflavonoid biosynthesis during seed development [15]. In the transient expression experiments of this study using tobacco leaves, the co-transfection of the *CHS8* gene with the effector construct demonstrated that the CHS8pro:GUS construct was activated by *GmMYBJ3*, and the GUS activity of the co-transfectant was 1.59-fold higher than that of the CHS8pro:GUS construct. In previous studies, *GmMYB176* was found to trans-activate the CHS8pro:GUS expression by 4.7-fold in *Arabidopsis* leaf protoplasts compared to the CHS8pro:GUS and the level of endogenous *CHS8* transcripts was increased by 169-fold in soybean protoplast within 48 h [41]; and *GmMYB12B2* trans-activated CHS8pro:GUS expression by 2-fold in soybean calli [42]. In this study, although *GmMYBJ3* activateed a lower expression level of *CHS8* than *GmMYB176* and *GmMYB12B2*, the co-transfection results indicate that *CHS8* is one of the target genes for *GmMYBJ3*.

This study has also shown that *GmMYBJ3* expresses in the tissues where isoflavonoids are synthesized in soybean. The expression of *GmMYBJ3* is most active in embryos and increases gradually as they develop, even it also expresses in other tissues including root, stem, leaf and floret. The correlation between *GmMYBJ3* expression level and total isoflavonoids accumulation further demonstrates that *GmMYBJ3* is involved in the isoflavonoid biosynthesis in soybean. Moreover, the roles of *GmMYBJ3* in regulating isoflavonoid biosynthesis have been further confirmed by the overexpression analysis in the transgenic plants. The results of significantly increased total seed isoflavonoid content and individual isoflavones, glycitin and genistin, but not for daidzin, genistein and daizein in the transgenic plants compared to the wild type provide a strong line of evidence in supporting the roles of *GmMYBJ3* in isoflavonoid biosynthesis. Because levels of total seed isoflavonoids were increased only by 1.5-fold in the soybean transgenic plants, which is only about half of those in the soybean seeds of the maize chimeric CRC gene transgenic plants [24], the function of *GmMYBJ3* is likely weaker than the maize CRC in regulating the phenylpropanoid pathway. Furthermore, C1 is a R2R3 MYB TF involved in anthocyanin accumulation in maize and must interact with R (bHLH TF) to be effective [43], whereas *AtMYB11*, *AtMYB12* and *AtMYB111* all can regulate flavonol synthesis by themselves [27,44]. Further study is required to determine whether *GmMYBJ3* needs to interact with other protein factors to enhance its function.

Previous study showed that overexpression of *LjMYB14* up-regulated expression of *PAL*, *C4H*, *4CL*, *IFS* and *IFR* genes in Lotus [34], and silencing *GmMYB176* down-regulated the expression level of *CHS8* and reduced the isoflavonoid content in soybean hairy roots [41]. We speculate that *GmMYBJ3* may affect soybean isoflavonoid biosynthesis through regulation of the structural genes for enzymes in the biosynthesis pathway. Of the twelve members in the CHI family, *CHI1A*, *PAL1* and *IFS2* are involved in seed isoflavonoid biosynthesis [35], and *CHS8* plays a crucial role in isoflavonoid biosynthesis [14,15]. Our analysis of the *PAL1*, *IFS2*, *CHS8* and *CHI1A* expression in the seeds of wild-type and transgenic plants indicates that *GmMYBJ3* does not have effect on *PAL1* and *IFS2*, but does have effect on *CHS8* and *CHI1A*. This result suggests that *GmMYBJ3* is able to activate the expression of *CHS8*. These results all indicate that *GmMYBJ3* participates in isoflavonoid biosynthesis by up-regulating the expression levels of *CHS8* and *CHI1A* in soybean seeds.

In conclusion, we have isolated a novel R2R3 MYB TF, *GmMYBJ3*, from soybean. *GmMYBJ3* encodes a nucleus-localized protein and has a transcription activation domain. *GmMYBJ3* activates the expression of *CHS8*, thus enabling to enhance the total and individual isoflavone contents, especially glycitin and genistin, in soybean seeds by up-regulating the genes encoding the enzymes involved in the isoflavonoid biosynthesis pathway.

## 4. Materials and methods

### 4.1 Plants materials

Soybean (*Glycine max* L.) cv. Williams 82 was used for expression analysis of *GmMYBJ3* in various soybean tissues. The cultivar was planted in the Crop Experimental Station of Jilin University near Changchun, China. Roots, stems and leaves were sampled between the ternate leaf stage and before flowering; flowers were sampled at the flowering stage; embryos were tagged on the first day of pollination and then collected on the 20th, 30th, 40th and 50th day after pollination and pod wall was separated from the 50th day embryo. All of the tissues were collected randomly from three independent plants, quickly frozen in liquid nitrogen and then stored at −80°C.

Cultivar Jilin 35 was used as the host plant for genetic transformation and overexpression analysis of *GmMYBJ3* in soybean. The T_2_ transgenic plants generated and wild-type Jilin 35 were grown in the transgenic controlled field, Jilin University. The tissues for expression analysis of the genes were collected from the transgenic plants as described above.

### 4.2 Isolation and sequence analysis of the *GmMYBJ3* gene

Isoflavonoid content QTL analysis allowed identification of a MYB TF (Glyma.06g193600) in the QTL region. Therefore, it was used an inquiry to search the soybean genome data (http://soybase.org/). A cDNA clone (GenBank accession number: KU664645) that is highly homologous to plant MYB TFs was identified and this cDNA clone was designated as *GmMYBJ3*. To reproduce the cDNA of the gene, total RNA was extracted from 50 d embryos of Williams 82 and reverse transcription polymerase chain reaction (RT-PCR) was performed using the primer pair, 5′- GAGCCCAAAGGGATCAAA -3′ (forward) and 5′- CGACCTCAAGTCCGCTAC -3′ (reverse) designed based on the cDNA sequence. The RT-PCR reaction contained 18.2 μL H_2_O, 1 μL of the first strand of cDNA, 1 μL (10 μM) of each primer, 2.5 μL of 10x PCR reaction buffer, 2 μL of dNTP mix and 0.3 μL of ExTaq DNA polymerase (Takara, China). The PCR reaction program was 95°C for 4 min, followed by 30 cycles (94°C for 40 s, 58°C for 40 s and 72°C for 80 s) and 72°C for 10 min at the final extension. The amplified fragments were cloned into the pMD-18T vector (Takara, China) and sequenced for confirmation. Phylogenetic analysis of the *GmMYBJ3* gene with foreign genes was performed using MEGA 7 with the Neighbor-Joining (NJ) algorithm [45].

### 4.3 Subcellular localization assay

The coding region of *GmMYBJ3* lacking a stop codon was ligated to the N-terminus of *GFP* to construct the pBI121-*GmMYBJ3-GFP* construct under the control of the cauliflower mosaic virus 35S (CaMV 35S) promoter in the pBI121 vector. This construct was then introduced into the *Agrobacterium tumefaciens* Strain EHA105 and used for subcellular localization analysis with transient transformation in onion epidermal cells. The *Agrobacterium* culture was prepared as described by Yang *et al*. [46]. The onion cells were dipped in prepared *Agrobacterium* solution for 30 min and then plated on Murashige and Skoog (MS) medium and incubated at 25°C in the dark for 16–48 h. Hoechst No.33342 was used to stain the nuclei of the onion cells and the transformed onion cells were observed using a confocal microscope (Olympus, Japan).

### 4.4 Transactivation analysis in the yeast GAL4 system

The *GmMYBJ3* coding sequence was cloned into the GAL4 DNA-BD binding domain in the pGBKT7 vector. The transactivation assay (PT3024-1) was performed as described by its manufacturer (Clontech, USA). The pGBKT7-*GmMYBJ3* plasmid was transformed into the yeast strain AH109 by the lithium acetate-mediated method [47], and the transformants were screened on SD/-Trp at 28°C for 2 d. Then, the transformants from SD/-Trp were streaked onto the solid medium SD/-Trp/-His/-Ade plus 3-AT. The plates were incubated for 3 d until they were used for a β-galactosidase assay. For the β-galactosidase assay, the transformant cells were imprinted onto Whatman filter paper and lysed by freezing with liquid nitrogen for 10 s and then thawing at room temperature. This process was repeated 3 times to completely lyse the cells. The filter was incubated in 2.5 ml of Z buffer containing 0.8 mg of 5-bromo-4-chloro-3-indolyl β-D-galactopyranoside supplemented with 21.51 g L^-1^ Na_2_HPO_4_·12 H_2_O, 6.22 g L^-1^ NaH_2_PO_4_·H_2_O, 0.75 g L^-1^ KCl, and 2.46 g L^-1^ MgSO_4_·7H_2_O at 30°C for 30 min to 8 h. The color reaction was monitored.

### 4.5 Transient expression

To construct the reporter plasmid, the 1577-bp *CHS8* promoter (F: 5’-AGCTGAGCAAGTATACCAACC-3’; R: 5’-GAGGTTGAAATGAAGGTGTGC-3’) was amplified by PCR and cloned into the pCAMBIA1301 plasmid to replace the CaMV 35S promoter. To construct the effector plasmid, the full coding region of *GmMYBJ3* was cloned into the pCB35SR1R2-GFP plasmid that was constructed as below and under the control of the CaMV 35S promoter. The reporter and the effector plasmids were introduced into the *Agrobacterium* EHA105 strain for further analysis. *Agrobacterium*-mediated transient expression was performed on the leaves of 4-week-old tobacco seedlings (*Nicotiana tabacum* cv. NC89) [46]. GUS activity analysis was performed after approximately 48 h [48]. All transfection experiments and GUS activity analysis were performed at least three times.

### 4.6 Quantitative real-time RT-PCR analysis

Total RNA was extracted from the soybean tissues sampled above using an RNAprep pure Plant Kit (Tiangen, China) and cDNA was synthesized using MMLV reverse transcriptase (Takara, China). Quantitative real-time RT-PCR analysis was performed with Applied Biosystems 7500 (Applied Biosystems, USA) using SYBR *Premix Ex Taq* (Takara, China). Each reaction contained 10 μL of SYBR Green I, 2 μL of cDNA samples, 0.4 μL of ROX Reference Dye II and 0.4 μL of 10 μM gene-specific primers for a final volume of 20 μL. The reaction was performed at 95°C for 30 s, followed by 40 cycles of 95°C for 5 s, 60°C for 34 s and 72°C for 30 s. Three biological replicates, with three technical replicates per biological replicate, were applied for the experiment. The soybean β-tubulin gene (GenBank accession No. GMU12286) was used as an internal control. The gene-specific primers used for real-time quantitative RT-PCR and their accession numbers are listed in Table 1. The data were analyzed using the comparative C_T_ method based on C_T_ values [49].

**Table 1:** Gene-specific primer pairs used for quantitative real time RT-PCR.

Gene name	Primer pairs	GenBank accession No.
*β-tubulin*	5’—GGAAGGCTTTCTTGCATTGGTA—3’	GMU12286
	5’—AGTGGCATCCTGGTACTGC—3’	
*GmMYBJ3*	5’—CGGTTCCCAGTAACACAGGT—3’	KU664645
	5’—TGATACCAGGTCGGAGATAGTTG—3’	
*GmCHS8*	5’—ATCCGCCAGGCACAAAGG—3’	NM_001317656
	5’—TGAAGTAGTAGTCAGGATAGGTGCT—3’	
*GmIFS2*	5’—AAGCCTCGTCTTCCCTTCATAG—3’	NM_001251586
	5’—CAAAGTAGAGAGAGAATAAGGGACC—3’	
*GmPAL1*	5’—TCAGAGTCAGCGAGAGAAGGAG—3’	XM_003521349
	5’—GGTGGTGACGCCGTAACTG—3’	
*GmCHI1A*	5’—TGTATCAGCGGCGGTCTTG—3’	AF276302
	5’—TCAATACCGCAGGCAATCG—3’	
*GmF3H*	5’—TTCATTGTCTCCAGCCATCTCC—3’	NM_001249868
	5’—CGCTGTATTCCTCAGTCACCG—3’	

### 4.7 Isoflavonoid analysis by high-performance liquid chromatography (HPLC)

Approximately 0.5 g of dry soybean seeds was ground, mixed with 10 mL of 80% methanol in distilled water followed by sonication for 20 min, and subsequently incubated at 80°C for 14 hours. The mixture was filtered through a 0.45-μm filter and transferred to 5-mL HPLC vials. Aliquots of this filtrate (20 μL) were utilized for HPLC analysis.

For HPLC, a Phenomenex C18 column (Shimadzu LC-20A, Japan, 250 mm × 4.6 mm, 5.0 μm) and binary gradient elution were used with solvent A (methanol, chromatography purity) and solvent B (distilled water). The ratio of solvent A was 15%–45% at 0–20 min, 45%–60% at 20–30 min, 60%–80% at 30–35 min, 80%–90% at 35–40 min, and 90%–15% at 40–45 min at a flow rate of 1 mL·min^−1^. The temperature of the column was maintained at 40°C and the detector wavelength was set at 254 nm. Isoflavones were identified by comparison with authentic standards of daidzein, genistein, daidzin, genistin and glycitin (Sigma, St. Louis, MO, USA), and quantification of the isoflavones was carried out by reference to an external standard.

### 4.8 Plasmid construction and plant transformation

The GATEWAY cloning technology was used to construct the plant expression vector [50]. First, the *GmMYBJ3* coding region was cloned into the entry vector pDONR221 (Invitrogen, USA) via a BP recombinant reaction. Second, the fragment in the entry vector was transferred to the destination vector, pCB35SR1R2-GFP, based on the LR recombinant reaction. The pCB35SR1R2-GFP-*GmMYBJ3* construct was heat shocked into *A*. *tumefaciens* EHA105 and then transformed into embryonic tips of soybean cv. Jilin 35. The transformed soybean embryonic tips were grown at 25°C/22°C with a light/dark cycle of 16 h light and 8 h darkness and a relative humidity of 60%. Transgenic T_0_ and T_1_ plants were cultured in a phytotron at 28°C/22°C with a light/dark cycle of 16 h light and 8 h darkness and a relative humidity of 60%.

### 4.9 Southern blot analysis

Southern blot analysis was performed to detect transgenic plants. DNA was extracted from the young leaves of T_2_ transgenic and wild-type plants (Jilin 35) using the CTAB method [51]. Approximately 10 μg of genomic DNA was digested with *Hin*d III (Takara, China). The digests were separated on a 0.8% agarose gel and blotted onto a Hybond-N+ nylon membrane (Roche Applied Science, Germany). The 501-bp PCR fragments (amplified with primer 5-ACCCACGTCATGCCAGTT-3 and 5’-CTAGGGGGATCTACCATG-3’) containing the bar gene (phosphinothricin acetyltransferase gene) coding region was labeled with Digoxigenin (DIG)-high prime and used as a probe for hybridization. Probe labeling, prehybridization, hybridization, membrane washing and signal detection were performed according to the manufacturer’s instruction (Roche Applied Science).

### 4.10 Statistical analysis

Statistical analysis was completed using the SPSS 21.0 program. Differences were defined at the two-tailed significance level of *P* ≤ 0.05.

## Supporting information

S1 Table The correlation between the *GmMYBJ3* expression level and total isoflavonoid content. * represent significant (P ≤ 0.05) correlation at the level of 2-tailed.
(DOCX)

## Abbreviations

3-AT: 3-amino-1, 2, 4-triazole
CaMV35S: cauliflower mosaic virus 35S
CHI1A: chalcone isomerase
CHS: chalcone synthase
cM: centimorgan
F3H: flavanone-3-hydroxylase
HPLC: high-performance liquid chromatography
IFS (2): isoflavone synthase (2)
ORF: open reading frame
PAL: phenylalanine ammonia-lyase
QTL (s): quantitative trait locus (loci)
RIL: recombining intercross line
TF(s): transcription factor(s)

## References

[1] Pueppke SG. The genetic and biochemical basis for nodulation of legumes by rhizobia. Crit Rev Biotechnol. 1996; 16(1):1–51. 10.3109/07388559609146599 8935908

[2] Ferguson BJ, Mathesius U. Signaling interactions during nodule development. Journal of Plant Growth Regulation. 2003; 22(1):47–72. 10.1007/s00344-003-0032-9

[3] Aoki T, Akashi T, Ayabe S-i. Flavonoids of leguminous plants: structure, biological activity, and biosynthesis. J Plant Res. 2000; 113(4):475–88. 10.1007/PL00013958

[4] Dixon RA, Achnine L, Kota P, Liu CJ, Reddy M, Wang L. The phenylpropanoid pathway and plant defence—a genomics perspective. Mol Plant Pathol. 2002; 3(5):371–90. 10.1046/j.1364-3703.2002.00131.x 20569344

[5] Dixon RA, Paiva NL. Stress-induced phenylpropanoid metabolism. The plant cell. 1995; 7(7):1085 10.1105/tpc.7.7.1085 12242399 PMC160915

[6] Gutierrez-Gonzalez JJ, Guttikonda SK, Tran L-SP, Aldrich DL, Zhong R, Yu O, et al Differential expression of isoflavone biosynthetic genes in soybean during water deficits. Plant and cell physiology. 2010; 51(6):936–48. 10.1093/pcp/pcq065 20430761

[7] Caldwell CR, Britz SJ, Mirecki RM. Effect of temperature, elevated carbon dioxide, and drought during seed development on the isoflavone content of dwarf soybean [Glycine max (L.) Merrill] grown in controlled environments. J Agric Food Chem. 2005; 53(4):1125–9. 10.1021/jf0355351 15713029

[8] Messina MJ. Legumes and soybeans: overview of their nutritional profiles and health effects. The American journal of clinical nutrition. 1999; 70(3):439s–50s. 10479216 10.1093/ajcn/70.3.439s

[9] Rochfort S, Panozzo J. Phytochemicals for health, the role of pulses. J Agric Food Chem. 2007; 55(20):7981–94. 10.1021/jf071704w 17784726

[10] Hahlbrock K, Scheel D. Physiology and molecular biology of phenylpropanoid metabolism. Annu Rev Plant Biol. 1989; 40(1):347–69. 10.1146/annurev.pp.40.060189.002023

[11] Koes RE, Spelt CE, van den Elzen PJ, Mol JN. Cloning and molecular characterization of the chalcone synthase multigene family of Petunia hybrida. Gene. 1989; 81(2):245–57. 10.1016/0378-1119(89)90185-6 2806915

[12] Todd JJ, Vodkin LO. Duplications that suppress and deletions that restore expression from a chalcone synthase multigene family. The Plant Cell. 1996; 8(4):687–99. 10.1105/tpc.8.4.687 12239396 PMC161129

[13] Tuteja JH, Vodkin LO. Structural features of the endogenous silencing and target loci in the soybean genome. Crop Sci. 2008; 48(Supplement_1):S-49–S-68. 10.2135/cropsci2007.10.0542tpg

[14] Dhaubhadel S, Gijzen M, Moy P, Farhangkhoee M. Transcriptome analysis reveals a critical role of CHS7 and CHS8 genes for isoflavonoid synthesis in soybean seeds. Plant Physiol. 2007; 143(1):326–38. 10.1104/pp.106.086306 17098860 PMC1761968

[15] Yi J, Derynck MR, Chen L, Dhaubhadel S. Differential expression of CHS7 and CHS8 genes in soybean. Planta. 2010; 231(3):741–53. 10.1007/s00425-009-1079-z 20016991

[16] Jung W, Yu O, Lau S-MC, O'Keefe DP, Odell J, Fader G, et al Identification and expression of isoflavone synthase, the key enzyme for biosynthesis of isoflavones in legumes. Nat Biotechnol. 2000; 18(2):208–12. 10.1038/72671 10657130

[17] Marles MS, Ray H, Gruber MY. New perspectives on proanthocyanidin biochemistry and molecular regulation. Phytochemistry. 2003; 64(2):367–83. 10.1016/S0031-9422(03)00377-7 12943753

[18] Stracke R, Werber M, Weisshaar B. The R2R3-MYB gene family in Arabidopsis thaliana. Curr Opin Plant Biol. 2001; 4(5):447–56. 10.1016/S1369-5266(00)00199-0 11597504

[19] Weisshaar B, Jenkins GI. Phenylpropanoid biosynthesis and its regulation. Curr Opin Plant Biol. 1998; 1(3):251–7. 10.1016/S1369-5266(98)80113-1 10066590

[20] Bommert P, Nagasawa NS, Jackson D. Quantitative variation in maize kernel row number is controlled by the FASCIATED EAR2 locus. Nat Genet. 2013; 45(3):334–7. 10.1038/ng.2534 23377180

[21] Huang X, Qian Q, Liu Z, Sun H, He S, Luo D, et al Natural variation at the DEP1 locus enhances grain yield in rice. Nat Genet. 2009; 41(4):494–7. 10.1038/ng.352 19305410

[22] Vadivel AKA, Sukumaran A, Li X, Dhaubhadel S. Soybean isoflavonoids: role of GmMYB176 interactome and 14-3-3 proteins. Phytochem Rev. 2016; 15(3):391–403. 10.1007/s11101-015-9431-3

[23] Gómez‐Maldonado J, Avila C, Torre F, Cañas R, Cánovas FM, Campbell MM. Functional interactions between a glutamine synthetase promoter and MYB proteins. The Plant Journal. 2004; 39(4):513–26. 10.1111/j.1365-313X.2004.02153.x 15272871

[24] Yu O, Shi J, Hession AO, Maxwell CA, McGonigle B, Odell JT. Metabolic engineering to increase isoflavone biosynthesis in soybean seed. Phytochemistry. 2003; 63(7):753–63. 10.1016/S0031-9422(03)00345-5 12877915

[25] Liu X, Yuan L, Xu L, Xu Z, Huang Y, He X, et al Over-expression of GmMYB39 leads to an inhibition of the isoflavonoid biosynthesis in soybean (Glycine max. L). Plant biotechnology reports. 2013; 7(4):445–55. 10.1007/s11816-013-0283-2

[26] Mellway RD, Tran LT, Prouse MB, Campbell MM, Constabel CP. The wound-, pathogen-, and ultraviolet B-responsive MYB134 gene encodes an R2R3 MYB transcription factor that regulates proanthocyanidin synthesis in poplar. Plant Physiol. 2009; 150(2):924–41. 10.1104/pp.109.139071 19395405 PMC2689947

[27] Mehrtens F, Kranz H, Bednarek P, Weisshaar B. The Arabidopsis transcription factor MYB12 is a flavonol-specific regulator of phenylpropanoid biosynthesis. Plant Physiol. 2005; 138(2):1083–96. 10.1104/pp.104.058032 15923334 PMC1150422

[28] Primomo VS, Poysa V, Ablett GR, Jackson C-J, Gijzen M, Rajcan I. Mapping QTL for individual and total isoflavone content in soybean seeds. Crop Sci. 2005; 45(6):2454–64. 10.2135/cropsci2004.0672

[29] Zeng G, Li D, Han Y, Teng W, Wang J, Qiu L, et al Identification of QTL underlying isoflavone contents in soybean seeds among multiple environments. Theor Appl Genet. 2009; 118(8):1455–63. 10.1007/s00122-009-0994-5 19266178

[30] Kassem MA, Meksem K, Iqbal M, Njiti V, Banz W, Winters T, et al Definition of soybean genomic regions that control seed phytoestrogen amounts. BioMed Research International. 2004; 2004(1):52–60. 10.1155/S1110724304304018 15123888 PMC545653

[31] Smallwood CJ. Detection of quantitative trait loci for marker-assisted selection of soybean isoflavone genistein. Master's Thesis, University of Tennessee, 2012.

[32] Gutierrez-Gonzalez JJ, Vuong TD, Zhong R, Yu O, Lee J-D, Shannon G, et al Major locus and other novel additive and epistatic loci involved in modulation of isoflavone concentration in soybean seeds. Theor Appl Genet. 2011; 123(8):1375–85. 10.1007/s00122-011-1673-x 21850478

[33] Park J-S, Kim J-B, Cho K-J, Cheon C-I, Sung M-K, Choung M-G, et al Arabidopsis R2R3-MYB transcription factor AtMYB60 functions as a transcriptional repressor of anthocyanin biosynthesis in lettuce (Lactuca sativa). Plant Cell Rep. 2008; 27(6):985–94. 10.1007/s00299-008-0521-1 18317777 PMC2413084

[34] Shelton D, Stranne M, Mikkelsen L, Pakseresht N, Welham T, Hiraka H, et al Transcription factors of Lotus: regulation of isoflavonoid biosynthesis requires coordinated changes in transcription factor activity. Plant Physiol. 2012; 159(2):531–47. 10.1104/pp.112.194753 22529285 PMC3375922

[35] Dastmalchi M, Dhaubhadel S. Soybean chalcone isomerase: evolution of the fold, and the differential expression and localization of the gene family. Planta. 2015; 241(2):507–23. 10.1007/s00425-014-2200-5 25385351

[36] Vom Endt D, Kijne JW, Memelink J. Transcription factors controlling plant secondary metabolism: what regulates the regulators? Phytochemistry. 2002; 61(2):107–14. 10.1016/S0031-9422(02)00185-1 12169302

[37] Su L-T, Wang Y, Liu D-Q, Li X-W, Zhai Y, Sun X, et al The soybean gene, GmMYBJ2. Acta Physiologiae Plantarum. 2015; 37(7):1–12. 10.1007/s11738-015-1889-5

[38] Cominelli E, Galbiati M, Vavasseur A, Conti L, Sala T, Vuylsteke M, et al A guard-cell-specific MYB transcription factor regulates stomatal movements and plant drought tolerance. Curr Biol. 2005; 15(13):1196–200. 10.1016/j.cub.2005.05.048 16005291

[39] Shirley BW, Kubasek WL, Storz G, Bruggemann E, Koornneef M, Ausubel FM, et al Analysis of Arabidopsis mutants deficient in flavonoid biosynthesis. The Plant Journal. 1995; 8(5):659–71. 10.1046/j.1365-313X.1995.08050659.x 8528278

[40] Weston K. Myb proteins in life, death and differentiation. Curr Opin Genet Dev. 1998; 8(1):76–81. 10.1016/S0959-437X(98)80065-8 9529609

[41] Yi J, Derynck MR, Li X, Telmer P, Marsolais F, Dhaubhadel S. A single‐repeat MYB transcription factor, GmMYB176, regulates CHS8 gene expression and affects isoflavonoid biosynthesis in soybean. The Plant Journal. 2010; 62(6):1019–34. 10.1111/j.1365-313X.2010.04214.x 20345602

[42] Li X-W, Li J-W, Zhai Y, Zhao Y, Zhao X, Zhang H-J, et al A R2R3-MYB transcription factor, GmMYB12B2, affects the expression levels of flavonoid biosynthesis genes encoding key enzymes in transgenic Arabidopsis plants. Gene. 2013; 532(1):72–9. 10.1016/j.gene.2013.09.015 24060295

[43] Grotewold E, Chamberlin M, Snook M, Siame B, Butler L, Swenson J, et al Engineering secondary metabolism in maize cells by ectopic expression of transcription factors. The Plant Cell. 1998; 10(5):721–40. 10.1105/tpc.10.5.721 9596632 PMC144024

[44] Stracke R, Ishihara H, Huep G, Barsch A, Mehrtens F, Niehaus K, et al Differential regulation of closely related R2R3‐MYB transcription factors controls flavonol accumulation in different parts of the Arabidopsis thaliana seedling. The Plant Journal. 2007; 50(4):660–77. 10.1111/j.1365-313X.2007.03078.x 17419845 PMC1976380

[45] Kumar S, Stecher G, Tamura K. MEGA7: Molecular Evolutionary Genetics Analysis version 7.0 for bigger datasets. Mol Biol Evol. 2016: msw054. 10.1093/molbev/msw054 27004904 PMC8210823

[46] Yang Y, Li R, Qi M. In vivo analysis of plant promoters and transcription factors by agroinfiltration of tobacco leaves. The Plant Journal. 2000; 22(6):543–51. 10.1046/j.1365-313x.2000.00760.x 10886774

[47] Gietz D, St Jean A, Woods RA, Schiestl RH. Improved method for high efficiency transformation of intact yeast cells. Nucleic Acids Res. 1992; 20(6):1425 1561104 10.1093/nar/20.6.1425 PMC312198

[48] Jefferson RA. Assaying chimeric genes in plants: the GUS gene fusion system. Plant molecular biology reporter. 1987; 5(4):387–405. 10.1007/BF02667740

[49] Livak KJ, Schmittgen TD. Analysis of relative gene expression data using real-time quantitative PCR and the 2− ΔΔCT method. methods. 2001; 25(4):402–8. 10.1006/meth.2001.1262 11846609

[50] Karimi M, Inzé D, Depicker A. GATEWAY^™^ vectors for Agrobacterium-mediated plant transformation. Trends Plant Sci. 2002; 7(5):193–5. 10.1016/j.plasmid.2015.06.003 11992820

[51] Porebski S, Bailey LG, Baum BR. Modification of a CTAB DNA extraction protocol for plants containing high polysaccharide and polyphenol components. Plant molecular biology reporter. 1997; 15(1):8–15. 10.1007/BF02772108

