# Supplementary material for: Isolation and characterization of GmMYBJ3, an R2R3-MYB transcription factor that affects isoflavonoids biosynthesis in soybean
Source: PLoS One. 2017 Jun 27;12(6):e0179990. doi: 10.1371/journal.pone.0179990 (PMC5487076; doi:10.1371/journal.pone.0179990)
Supplement: S1 Table — * represent significant (P ≤ 0.05) correlation at the level of 2-tailed. (DOCX) [file pone.0179990.s001.docx]

**S1 Table.** **The correlation between the *GmMYBJ3* expression level and total isoflavonoid content**.

|  | | total isoflavonoid content | relative GmMYBJ3 expression level |
| --- | --- | --- | --- |
|  | Pearson Correlation | 1 | .674* |
| total isoflavonoid content | Sig. (2-tailed) |  | .046 |
|  | N | 9 | 9 |
|  | Pearson Correlation | .674* | 1 |
| relative GmMYBJ3 expression level | Sig. (2-tailed) | .046 |  |
|  | N | 9 | 9 |

^1^ * represent significant (P ≤ 0.05) correlation at the level of 2-tailed.
